# Role of Bruton’s Tyrosine Kinase in Stage III Colorectal Cancer

**DOI:** 10.3390/cancers11060880

**Published:** 2019-06-24

**Authors:** Debora Basile, Lorenzo Gerratana, Angela Buonadonna, Silvio Ken Garattini, Tiziana Perin, Emanuela Grassilli, Gianmaria Miolo, Maria Grazia Cerrito, Claudio Belluco, Giulio Bertola, Antonino De Paoli, Renato Cannizzaro, Marialuisa Lavitrano, Fabio Puglisi, Vincenzo Canzonieri

**Affiliations:** 1 Department of Medicine (DAME), University of Udine, 33100 Udine, Italy; deborabasile1090@gmail.com (D.B.); gerratana.lorenzo@spes.uniud.it (L.G.); silvioken.garattini@cro.it (S.K.G.); fabio.puglisi@cro.it (F.P.); 2 Department of Medical Oncology, Centro di Riferimento Oncologico di Aviano (CRO), IRCCS, 33081 Aviano, Italy; abuonadonna@cro.it (A.B.); gmiolo@cro.it (G.M.); 3 Pathology Unit, Centro di Riferimento Oncologico di Aviano (CRO), IRCCS, 33081 Aviano, Italy; tperin@cro.it; 4 CRO Biobank, Centro di Riferimento Oncologico di Aviano (CRO), IRCCS, 33081 Aviano, Italy; 5 Department of Medicine and Surgery, University of Milano-Bicocca, 33081 Milan, Italy; emanuela.grassilli@unimib.it (E.G.); mariagrazia.cerrito@unimib.it (M.G.C.); marialuisa.lavitrano@unimib.it (M.L.); 6 Surgical Oncology, Centro di Riferimento Oncologico di Aviano (CRO), IRCCS, 33081 Aviano, Italy; cbelluco@cro.it (C.B.); gbertola@cro.it (G.B.); 7 Radiation Oncology, Centro di Riferimento Oncologico di Aviano (CRO), IRCCS, 33081 Aviano, Italy; adepaoli@cro.it; 8 Division of Oncological Gastroenterology, Centro di Riferimento Oncologico di Aviano (CRO), IRCCS, 33081 Aviano, Italy; rcannizzaro@cro.it; 9 Department of Medical, Surgical and Health Science, University of Trieste School of Medicine, 34100 Trieste (TS), Italy

**Keywords:** colon cancer, Bruton’s tyrosine kinase, BTK

## Abstract

Background: Bruton’s tyrosine kinase (BTK) is involved in the immune response and its deficiency impairs B cell maturation. We evaluated the expression of a novel BTK isoform, p65BTK, in colorectal cancer (CRC), to identify its impact on survival. Materials and Methods: This retrospective study evaluated 87 consecutive stage III CRC patients treated at the National Cancer Institute of Aviano (1999–2017). Multiple specimens were collected and analyzed for staining intensity and percentage of tumor cells positive for p65BTK. Prognostic impact was tested by univariate Cox regression analysis. Results: After a median follow-up of 82.59 months, median disease-free survival (DFS) and overall survival (OS) were 11.67 months and 31.33 months, respectively. Interestingly, 10% of patients did not express p65BTK. For the immunohistochemistry IHC intensity 1, the best cutoff point was 1% of p65BTK positivity; for IHC intensity 2, it was 50%; and for IHC intensity 3, it was 80%. Through univariate analysis, patients with highly expressed p65BTK (IHC intensity 3 and ≥80%) were shown to have the worst prognosis in terms of DFS (HR: 6.23; *p* = 0.005; 95% C.I. 1.75–22.79) and OS (HR: 2.54; *p* = 0.025; 95% C.I. 1.12–5.76). Conclusions: p65BTK is frequently expressed in CRC and, if highly expressed, is an unfavourable prognostic factor. However, further confirmation is needed and its potential targeting needs to be studied.

## 1. Introduction

Colorectal cancer (CRC) is the third most frequently diagnosed cancer and the fourth cause of cancer-related death worldwide [1,2]. Breakthroughs in cancer biology are currently changing the therapeutic landscape in many cancer types, including CRC. Recently, molecular biology was also demonstrated to be a cornerstone evaluation for CRC, highlighting the heterogeneity of this neoplasm [3,4]. Namely, it has been shown that specific gene alterations have both a prognostic and/or predictive value in CRC, with important implications for clinical practice. Therefore, the aggressiveness of CRC has been defined not only by its clinico-pathological characteristics, but also by its molecular and immunological profile [5,6,7,8,9]. Despite novel therapeutic approaches having recently reshaped the overall strategy of treating metastatic CRC, there is still a need to identify factors able to predict the risk of recurrence in order to help clinicians in the choice of a more intensive adjuvant treatment.

In this scenario a new potential biomarker is emerging, i.e., Bruton’s tyrosine kinase (BTK), which could play a key role in identifying resistant tumors to conventional adjuvant treatment [10]. The BTK gene is located at Xq22.1 and its product was first described as the deficient protein involved in the development of X-linked agammaglobulinemia. BTK is a non-receptor tyrosine kinase belonging to the TEC family, the second largest family of cytoplasmic tyrosine kinases whose other members are TEC (tyrosine kinase expressed in hepatocellular carcinoma), BMX (bone-marrow tyrosine kinase gene on chromosome X), ITK (interleukin-2 (IL-2)-inducible T-cell kinase), and RLK (resting lymphocyte kinase). Most members of the family are variously expressed in the hematopoietic lineage and in solid tissues, both normal and tumoral, and are involved in the intracellular signaling mechanisms of cytokine receptors, lymphocyte surface antigens, heterotrimeric G-protein coupled receptors, and integrin molecules [11,12]. For instance, for a long time, BTK has been thought to only be expressed in the hematopoietic lineage; in particular, it is constitutively expressed in B cells, where it is activated downstream of the B cell receptor (BCR) and therefore plays a key role in promoting the maturation, differentiation, and proliferation of B lymphocytes. In fact, BTK deficiency impairs B cell maturation and antibody production. Furthermore, BTK releases anti-apoptotic signals (NF-kB, PI3K/AKT/mTOR pathway) to sustain lymphocytes activation [13] and is therefore involved in the innate and adaptive immune response. In mature differentiated B lymphocytes, its transcription is downregulated, whereas it is not expressed at all in T cells [10,12]. Considering the critical role of BTK in several B-cell malignancies, a BTK-specific inhibitor, ibrutinib, is currently used to treat some types of lymphoma (such as mantle cell lymphoma) and chronic lymphocytic leukemia [13].

More recently, some studies showed BTK expression in several solid tumors [12]. In particular, in CRC, a new isoform, p65BTK, is abundantly expressed, through hnRNPK-dependent and IRES-driven translation, from mRNA containing an alternative first exon in the 5′UTR. In addition, p65BTK is post-transcriptionally regulated, via hnRNPK, by the mitogen-activated protein kinase (MAPK) pathway. It is endowed with strong transforming activity that depends on ERK1/2 and its inhibition abolishes RAS transforming activity. Accordingly, p65BTK overexpression in colon cancer tissues correlates with extracellular-signal-regulated kinase 1/2 (ERK1/2) activation and its inhibition affects the growth and survival of colon cancer cells [14]. Moreover, its inhibition re-sensitizes drug-resistant colon cancer cell lines, organoids, and xenografts to 5-Fluorouracil. Therefore, this could suggest that BTK could be a potential new target in CRC treatment [14]. The aim of this study is to evaluate the expression of p65BTK in nodes positive for CRC and to analyze its prognostic role in terms of overall survival (OS) and disease-free survival (DFS).

## 2. Results

### 2.1. Clinico-Pathological Characteristics

The present study included 87 patients with a diagnosis of pathological stage T3 and T4 nodes positive for CRC (patients’ characteristics are shown in Table 1). In the whole cohort, 70% of patients were less than 70 years old. About 57% of them had right colon carcinoma and 77% had a pT3 node positive tumor. As for the molecular biology, in 20% of patients, KRAS analysis was performed, and about 47% of them had a mutation, mainly in exon 2. Overall, 61% of patients received adjuvant treatment and, in 67% of them, the regimens adopted included capecitabine and oxaliplatin or 5-fluorouracil and oxaliplatin.

A percentage of patients did not receive adjuvant treatment because of the patient decision or rapidly progressive disease. Of note, 29% of patients had a metastatic disease and 84% of them received first-line chemotherapy.

### 2.2. Expression Analysis

**Figure 1:**
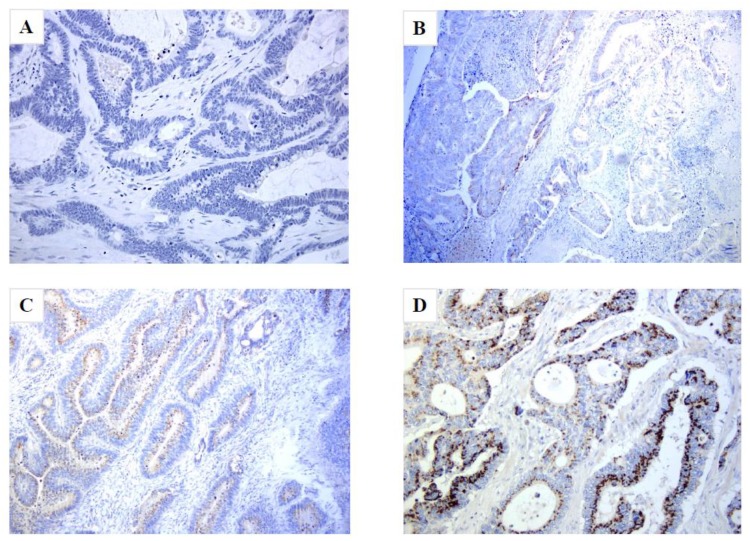
p65BTK expression in specimen samples of stage III CRC. p65BTK *Hematoxylin* and *Eosin* (H&E) staining, original magnification 200×. (**A**) Tumor sample with 0% p65BTK expression. (**B**,**C**) Tumor samples with an intermediate percentage of expression. (**D**) Tumor sample with 100% p65BTK expression.

Interestingly, 10% of patients did not express p65BTK. About 54% of patients expressed p65BTK positivity in more than 90% of peritumoral healthy tissue, and the median percentage expressed in peritumoral healthy tissue was 71%. Moreover, 46% of patients expressed an intensity score of 3 in peritumoral healthy tissue. Figure 1.

**Figure 2:**
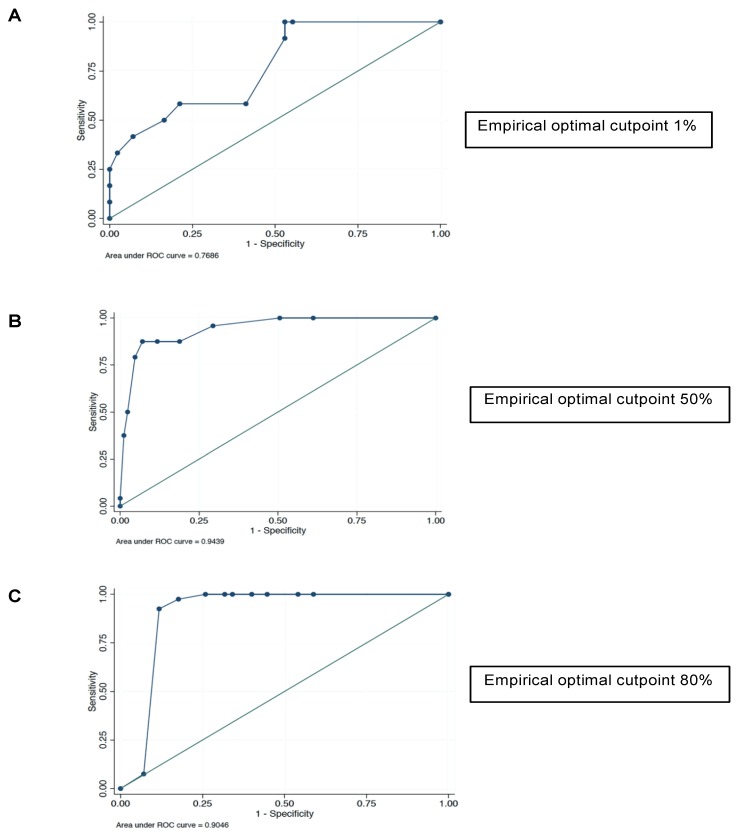
Receiver operating characteristic (ROC) curve analysis used to identify the optimal cutoff point capable of detecting a tumor-derived sample. (**A**) Immunohistochemistry (IHC) intensity 1: the threshold identified was 1% p65BTK positivity. (**B**) IHC intensity 2: the threshold identified was 50% p65BTK positivity. (**C**) IHC intensity 3: the threshold identified was 80% p65BTK positivity.

The best cutoff point capable of detecting a tumor-derived sample was calculated through Liu’s method and a 60% BTK positivity threshold was identified (area under the curve: 0.75). Therefore, the best cutoff point capable of detecting a tumor-derived sample was also calculated according to the intensity score. In the class with IHC intensity 1, the threshold identified was 1% of p65BTK positivity (area under the curve: 0.74) (Figure 2A); in the class with IHC intensity 2, the threshold identified was 50% of p65BTK positivity (area under the curve: 0.90) (Figure 2B); and in the class with IHC intensity 3, the threshold identified was 80% of p65BTK positivity (area under the curve: 0.90) (Figure 2C). Factors associated with BTK expression are shown in Table 2. Moreover, treatment received and disease status have been reported for patients with BTK immunohistochemistry (IHC) ≥ 80% and intensity 3 in Table 3.

### 2.3. Survival Analysis

After a median follow-up of 82.59 months, the median DFS was 11.67 months (25–75th percentiles: 5.00–17.42 months), whereas OS was 31.33 months (25–75th percentiles: 10.88–45.3 months).

**Figure 3:**
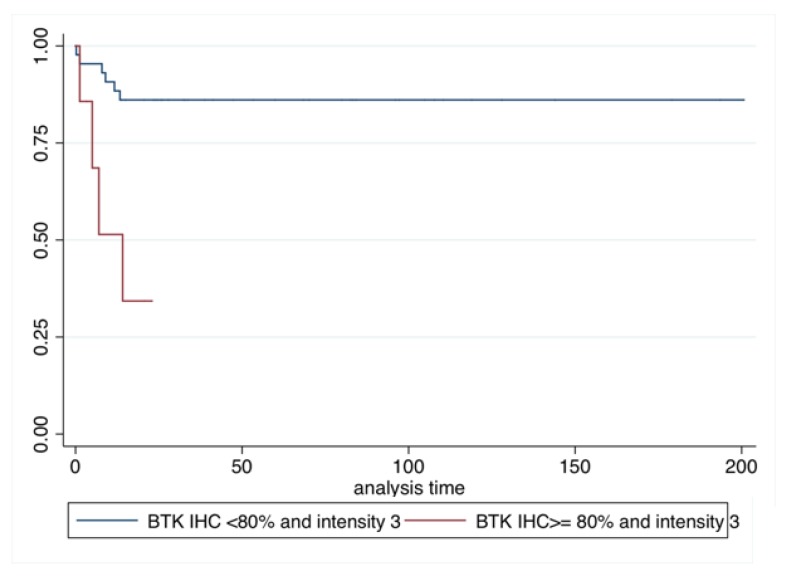
Kaplan–Meier disease-free survival (DFS).

**Figure 4:**
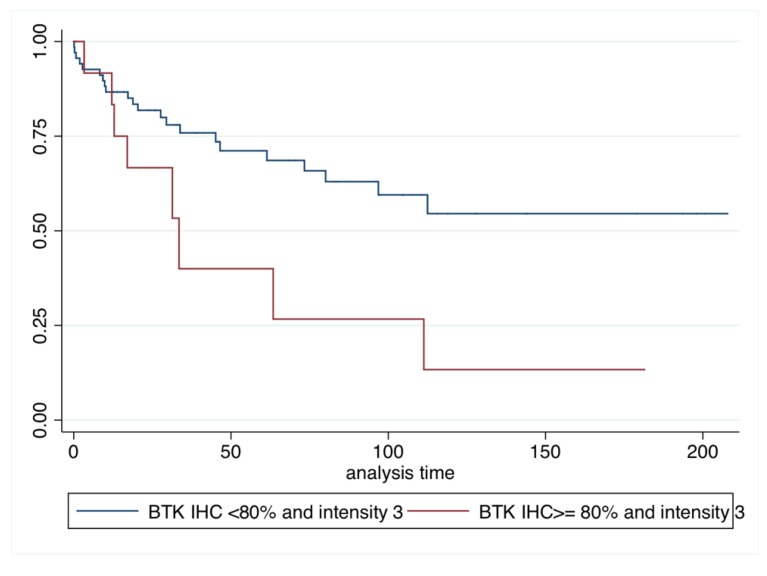
Kaplan–Meier overall survival (OS).

By conducting univariate analysis, we showed that patients with only p65BTK IHC intensity 3 and a positivity ≥80% had the worst prognosis in terms of DFS (HR: 6.23; *p* = 0.005; 95% C.I. 1.75–22.79) (Table 4 and Figure 3) and OS (HR: 2.54; *p* = 0.025; 95% C.I. 1.12–5.76) (Table 5 and Figure 4).

### 2.4. Exploratory Analysis

**Figure 5:**
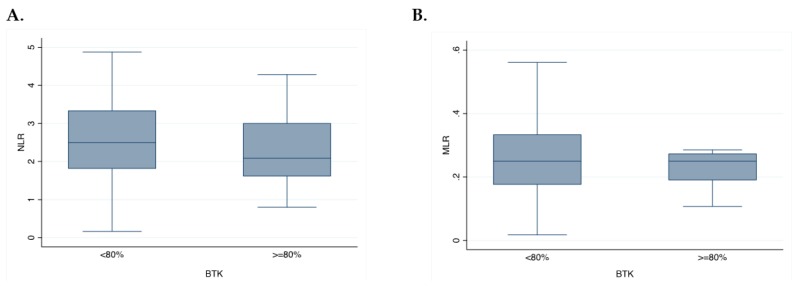
Exploratory analysis. (**A**) Box representing the association of neutrophils-to-lymphocytes (NLR) with BTK intensity 3. (**B**) Box representing the association of monocytes-to-lymphocytes (MLR) with BTK intensity 3.

To explore potential circulating biomarkers, such as the inflammation index (NLR) and immune-suppressive index (MLR), we tested the association with p65BTK expression. Of note, no association was observed with NLR (*p* = 0.15) and MLR (*p* = 0.39). Similarly, no association was found between p65BTK expression and KRAS mutation (*p* = 0.93) (Figure 5A,B).

## 3. Discussion

In recent years, several therapeutic strategies have improved the prognosis of patients with metastatic CRC. However, not all patients seem to benefit from systemic chemotherapy, probably due to primary or acquired drug resistance [15,16]. Therefore, the study of new molecular targets could allow the identification of new prognostic and predictive biomarkers and the development of new therapeutic approaches.

Interestingly, in the last few years, CRC has been classified in consensus molecular subtypes (CMS): CMS1 (microsatellite instable tumors), CMS2 (chromosomal instable tumors), CMS3 (KRAS mutated tumors), and CMS4 (cancer with mesenchymal characteristics) [6,17]. Intriguingly, microsatellite instability (MSI) tumors have a significantly high mutational burden, mainly due to mismatch–repair mechanism deficiency, which leads to the expression of a higher amount of non-self antigens and a consequent stimulation of the immune response [18].

In the present study, we analyzed the expression of p65BTK, a novel BTK isoform, in a cohort of CRC patients in order to define its role as a prognostic factor in patients with stage III disease. Due to its engagement following BCR activation, BTK is essential for B-cell differentiation and proliferation [19]. Moreover, BTK is activated along many other signaling pathways triggered in B cells, including those downstream of the chemokine receptors, Toll-like receptors, and Fc receptor. In addition, BTK plays a key role in the pathway downstream of receptor activator of nuclear factor κB (RANK) in osteoclasts, in collagen and CD32 signaling in platelets, and in the NLRP3 inflammasome in macrophages and neutrophils [12,19,20]. Although BTK was described as a kinase expressed only in bone-marrow-derived cells, more recently, BTK expression has also been detected in various solid tumors, such as those of ovarian cancer, CRC, prostate cancer, and brain cancer, with BTK overexpression being associated with a worse prognosis in the latter [12,21]. Moreover, a recent study identified p65BTK, a novel oncogenic isoform, expressed in CRC cell lines and tissues, showing a remarkable ability in transformation induction [10].

Our study showed that most tumor tissues expressed p65BTK and approximately 41% of samples expressed an IHC intensity of 3. Through Liu’s method, we identified a cut-off of 1% for tumors with intensity 1, 50% for tumors with intensity 2, and 80% for tumors with intensity 3. More interestingly, patients with the p65BTK isoform with intensity 3 and an IHC expression ≥80% had a worse survival both in terms of DFS (HR: 6.23; *p* = 0.005; 95% C.I. 1.75–22.79) and OS (HR: 2.54; *p* = 0.025; 95%C.I. 1.12–5.76), by univariate analysis. It was not possible to perform a multivariate analysis because no factors were significantly associated with prognosis in the univariate analysis. Based on these preliminary data, the p65BTK isoform could be a prognostic factor used to identify patients with a worse outcome and could be a potential target for treatment with BTK inhibitors to enhance the chemotherapy effect.

Notably, the expression levels of the p65BTK isoform are regulated downstream of the RAS/MAPK pathway: we previously demonstrated that in colon cancer, p65BTK expression parallels ERK-1/2 activation and is downregulated by ERK inhibitors. Moreover, p65BTK expression is induced in NIH-3T3 cells following the overexpression of activated RAS [10]. We recently confirmed that, in NSCLC, p65BTK expression is also regulated by the activation of the RAS/MAPK pathway [10,22]. In the present study, no association was observed with RAS mutation (*p* = 0.93), probably due to the low number of patients analyzed for RAS mutation.

Several efforts clarified the role of the immune system in regulating cancer growth [23]. Innate immune system cells (e.g., macrophages, neutrophils, myeloid derived suppressor cells, mast cells, eosinophils, antigen-presenting cells and adaptive immune cells such as T and B lymphocytes and, natural killer (NK) cells are involved [24,25]. In vitro studies, as well as experimental animal models, have led to a better understanding of the mechanism regulating the tumor microenvironment. During clonal selection, cancer cells develop several mechanisms to avoid and hijack immune effectors, thus creating an immunosuppressive microenvironment [23]. As previously reported, BTK plays a crucial role in the immune-suppressive state of the tumor [12,26,27,28,29]. Recent studies showed that in vivo ibrutinib treatment depletes myeloid-derived suppressor cells in tumor-bearing mice and prevents the secretion of immune suppressive cytokines. Moreover, in vitro studies showed a high level of CD8+ T lymphocytes after 3 days of treatment with ibrutinib [30]. Furthermore, ibrutinib reduced the production of TNFα, IL1β, and MCP-1 by macrophages and monocytes. Moreover, it decreased mast cells degranulation by interfering with the mechanisms of Ig-E secretion, suggesting that ibrutinib reverses the immune suppressive microenvironment. As a consequence, the reduction of peri-tumoral fibrosis and collagen deposition was observed, together with a decrease in the tumor vasculature density required for the tumor cell survival [12,31]. These histological features are most expressed in patients with recurrent disease and are associated with a worse prognosis [32].

Therefore, the use of BTK inhibitors could result in anti-cancer activity not only in hematological malignancies, but also in solid tumors [18,20], both indirectly, via acting on the tumor microenvironment (TME), and directly, by acting on the tumor cells themselves [11,12]. High NLR and MLR are indirect biomarkers of TME and are respectively associated with systemic inflammation and immune suppression [33,34,35]. Therefore, in the present study, we exploratively evaluated the association between p65BTK expression and these indexes performed before the surgery. However, no association was observed between p65BTK and NLR or MLR, probably due to the small sample size of the study and its retrospective nature. Due to these interesting observations, the future directions of our work will be focused on a more precise definition of the crosstalk between p65BTK and specific immune cells (e.g., lymphocytes infiltration, macrophages polarization). Furthermore, we will explore the role of this molecule in light of the better knowledge of the all-RAS mutational status of our patients.

## 4. Material and Methods

### 4.1. Study Design

This observational, retrospective, no-profit, monocentric cohort study examined data of 87 consecutive patients with stage III (pT3-pT4 with positive lymph-nodes) CRC treated at the National Cancer Institute of Aviano, Italy, from January 1999 to December 2017. All patients had a confirmed histological diagnosis of CRC. Informed consent was obtained for the use of clinical data, rendered anonymous, for purposes of clinical research, epidemiology, training, and the study of diseases.

The study was conducted in accordance with the Declaration of Helsinki, and the protocol was approved by the Institutional Review Board of the National Cancer Institute of Aviano and by Ethic Committee (CEUR) (Number of protocol CRO-2019-35). Data were obtained from electronic medical records according to strict privacy standards. The study aimed to evaluate the expression of p65BTK in nodes positive for CRC and to estimate the role of BTK in foreseeing the outcome of patients with stage III CRC. Moreover, the association between BTK and a patient’s immunological profile was explored through an analysis of the neutrophil-to-lymphocytes ratio (NLR) and monocyte-to-lymphocytes ratio (MLR). DFS was defined as the time from surgery to the first evidence of recurrence. OS was defined as the time between surgery and death from any cause.

### 4.2. Tissue Samples

Multiple specimens from surgical samples of 87 consecutive patients with stage III CRC were collected by a pathologist. Formalin-fixed, paraffin-embedded specimens (matched healthy peritumoral/tumoral tissues from the same patient) were used to perform pathological characterization and p65BTK evaluation by immunohistochemistry (IHC).

### 4.3. Immunohistochemistry Assessment of BTK

Specimens were processed and embedded in paraffin. Whole tissue samples were stained with *Hematoxylin* and *Eosin* (H&E) using standard IHC procedures to perform pathological evaluation. On p65BTK specimens, the staining intensity and percentage of positive tumor cells were defined as follows: negative/weak 1+, moderate 2+, or strongly positive 3+, and percentage of expression, respectively.

### 4.4. Anti-p65BTK Antibody Production and Characterization

Informative data about anti-p65BTK antibody production and characterization were evaluated in a previous study conducted on another CRC cohort [36]. Briefly, BN30 polyclonal antibodies were obtained in rabbits by immunization with the N-terminal decapeptide of p65BTK, conjugated to keyhole limpet hemocyanin via an additional C-terminal cysteine residue and validated as follows: specificity of the BN33 antiserum (IgG fraction), used for the enzyme-linked immunosorbent (ELISA) assay, was assessed by western blot analysis on lysate HCT116p53KO cells transfected with a control (luc) or p65BTK-specific siRNA and by ELISA on the same lysates, as well as purified recombinant p77BTK. The specificity of BN30 polyclonal antiserum (IgG fraction), used for IHC, was assessed by western blot analysis on lysate SW480 cells transfected with a control (luc) or p65BTK-specific siRNA and by IHC, on sections from cell blocks of SW480 p65BTK-expressing and p65BTK-silenced cells.

### 4.5. Blood Sample Analysis

NLR and MLR were determined as the absolute neutrophil count divided by the absolute lymphocyte count, and the absolute monocyte count divided by the absolute lymphocyte count, respectively. White blood cells’ data were used for the analysis if the blood samples had been obtained within 1 month before the start of first-line chemotherapy.

### 4.6. Statistical Analysis

Patients’ clinico-pathological characteristics were summarized through a descriptive analysis. Categorical variables were reported as the frequency distribution, whereas continuous variables were reported as the median value and range. The association of BTK with indirect inflammation and immune biomarkers (LDH and MLR) was explored by the Wilcoxon rank-sum test or Kruskal–Wallis test, as statistically appropriate. Prognostic factors in terms of OS were tested in a univariate model by Cox regression with a 95% confidence interval (95% C.I.). Differences in survival were tested by a log-rank test and represented by Kaplan–Meier survival curves. A receiver operating characteristic (ROC) analysis was performed to identify the optimal cutoff point capable of detecting a tumor-derived sample (Liu’s method) [37]. Factors associated with BTK expression were investigated through Fisher’s exact test and a Chi-squared test, as appropriate.

A two-sided *p* < 0.05 was considered statistically significant. Statistical analysis was performed with STATA (StataCorp, www.stata.com (2015) Stata Statistical Software: Release 14.2. College Station, TX: StataCorp LP).

## 5. Conclusions

The present study showed that p65BTK is frequently expressed in CRC and, if highly expressed (IHC ≥ 80% and intensity 3), it has an unfavourable impact on prognosis in terms of DFS and OS. In recent years, several anti-cancer treatments have improved CRC prognosis. However, not all patients seem to benefit from systemic chemotherapy, probably due to primary or acquired drug resistance. Therefore, it is essential to identify CRC patients who would mostly benefit from enhanced adjuvant chemotherapy. In this landscape, the p65BTK isoform seems to represent a potential target for new enhanced targeted therapy. However, further studies are needed to confirm these preliminary data.

## Figures and Tables

**Table 1:** Patients’ characteristics.

Variables	*N* = 87	%
**Age**		
<70	61	70.11
≥70	26	29.89
**Sex**		
M	51	58.62
F	36	41.38
**Sidedness**		
Right	50	57.47
Left	37	42.53
**Grading**		
G2	65	74.71
G3	22	25.29
**Histology**		
Adenocarcinoma	77	88.51
Mucinous adenocarcinoma	10	11.49
**Adj CT**		
No	25	28.73
Yes	53	60.91
**PD (metastatic disease)**		
No	43	49.42
Yes	25	28.73
**Rapid disease**		
No	75	86.21
Yes	12	13.79
**KRAS**		
WT	9	10.34
Mut	10	11.49
**BRAF**		
WT	13	14.94
Mut	3	3.44
**BTK 0%**	9	10.34
**BTK IHC ≥ 1% and intensity 1**		
No	38	43.68
Yes	47	54.02
Missing (lack of sample)	2	2.30
**BTK IHC ≥ 50% and intensity 2**		
No	75	86.2
Yes	10	11.5
Missing (lack of sample)	2	2.30
**BTK IHC ≥ 80% and intensity 3**		
No	70	80.45
Yes	15	17.25
Missing (lack of sample)	2	2.30

**Table 2:** Bruton’s tyrosine kinase (BTK) expression and clinico-pathological parameters.

Variables	BTK Intensity 1			BTK Intensity 2			BTK Intensity 3		
	IHC < 1%	IHC ≥ 1%	*p*	IHC < 50%	IHC ≥ 50%	*p*	IHC < 80%	IHC ≥ 80%	*p*
	*N* = 38	*N* = 47		*N* = 75	*N* = 11		*N* = 70	*N* = 15	
**Sidedness**									
Right	23	26	0.629	42	7	0.400	42	7	0.343
Left	15	21		33	3		28	8	
**Grading**									
G2	20	26	0.813	*38*	*8*	0.083	40	6	0.977
G3	6	9		*15*	*0*		13	2	
**Histotype**									
Adenocarcinoma	21	27	0.732	44	4	**0.034**	40	8	0.114
Mucinous adenocarcinoma	5	8		9	4		13	0	
**KRAS**									
Wild type	5	4	0.498	37	6	0.533	39	4	0.161
Mutated	4	6		21	2		18	5	
**Metastatic disease**									
No	16	27	0.241	8	1	0.596	8	1	0.937
Yes	12	11		8	2		9	1	
**Age**									
<70	28	30	0.403	49	9	0.127	48	10	0.826
≥70	10	16		25	1		21	5	
**Sex**									
M	21	30	0.423	47	4	0.169	42	9	1.000
F	17	17		28	6		28	6	

Values in bold are statistically significant.

**Table 3:** Bruton’s tyrosine kinase (BTK) immunohistochemistry (IHC) ≥ 80% and intensity 3: treatment received and disease status.

Variables	*N* = 15	%
**OS**		
Uncensored	9	60.00
Censored	6	40.00
**Rapid disease**		
Yes	3	20.00
No	12	80.00
Metastatic recurrence		
Yes	5	33.00
No	4	26.66
Missing	6	40.00
**Adj chemotherapy received**		
Fluoropyrimidenes (only)	3	20.00
Fluoropyrimidines plus oxaliplatin	6	40.00
Missing	6	40.00

**Table 4:** Univariate analysis of disease-free survival (DFS).

Variables	HR	*p*	95%CI
**Age**			
≥70	0.81	0.753	0.21–2.99
**Sidedness**			
Left	1.18	0.769	0.37–3.74
**KRAS**			
Mut	0.31	0.210	0.05–1.92
**BRAF**			
Mut	1.33	0.814	0.11–15.06
**BTK IHC ≥ 1% and intensity 1**			
Yes	0.71	0.601	0.20–2.48
**BTK IHC ≥ 50% and intensity 2**			
Yes	1.83	0.953	0.52–4.65
**BTK IHC ≥ 80% and intensity 3**			
**Yes**	**1.18**	**0.005**	**1.75–22.79**

Values in bold are statistically significant.

**Table 5:** Univariate analysis of overall survival (OS).

Variables	HR	*p*-Value	95%CI
**Age**			
≥70	1.49	0.278	0.72–3.11
**Sidedness**			
Left	0.95	0.910	0.46–1.96
**KRAS**			
Mut	0.39	0.213	0.09–1.69
**BRAF**			
Mut	7.59	0.099	0.68–84.41
**BTK IHC ≥ 1% and intensity 1**			
Yes	0.61	0.190	0.30–1.26
**BTK IHC ≥ 50% and intensity 2**			
Yes	0.89	0.843	0.31–2.58
**BTK IHC ≥ 80% and intensity 3**			
**Yes**	**2.54**	**0.025**	**1.12–5.76**

Values in bold are statistically significant.
